# Pollen-Mediated Gene Flow in Maize: Implications for Isolation Requirements and Coexistence in Mexico, the Center of Origin of Maize

**DOI:** 10.1371/journal.pone.0131549

**Published:** 2015-07-10

**Authors:** Baltazar M. Baltazar, Luciano Castro Espinoza, Armando Espinoza Banda, Juan Manuel de la Fuente Martínez, José Antonio Garzón Tiznado, Juvencio González García, Marco Antonio Gutiérrez, José Luis Guzmán Rodríguez, Oscar Heredia Díaz, Michael J. Horak, Jesús Ignacio Madueño Martínez, Adam W. Schapaugh, Duška Stojšin, Hugo Raúl Uribe Montes, Francisco Zavala García

**Affiliations:** 1 Monsanto Company, 800 North Lindbergh Blvd, St. Louis, Missouri, 63167, United States of America; 2 Instituto Tecnológico de Sonora, Dirección de Recursos Naturales, 5 de Febrero 818 Sur, Colonia Centro Cd. Obregón, Sonora, C.P. 85000, México; 3 Universidad Autónoma Agraria Antonio Narro, Unidad Laguna, Periférico Raúl López Sánchez y Carretera Santa Fe, Col. Valle Verde, Torreón, Coahuila, C.P. 27059, México; 4 Monsanto Comercial, Park Plaza Torre II, piso 1. Ave. Javier Barros Sierra #504, Col. Santa Fe, Del. Álvaro Obregón, México D.F., CP 01210, México; 5 Universidad Autónoma de Sinaloa, Facultad de Ciencias Químico Biológicas, Ave. Las Américas y Josefa Ortiz, S/N Culiacán, Sinaloa, C.P. 80000, México; 6 Universidad Autónoma de Chihuahua, Facultad de Ciencias Agrícolas y Forestales, Km. 2.5 Carr. Delicias-Rosales, Cd. Delicias, Chihuahua, C.P. 33000, México; 7 Universidad Autónoma de Nuevo León, Facultad de Agronomía, Av. Francisco Villa S/N, Col. Ex Hacienda "El Canadá," Escobedo, Nuevo León, C.P. 66050, México; Instituto de Biología Molecular y Celular de Plantas (IBMCP), SPAIN

## Abstract

Mexico, the center of origin of maize (*Zea mays* L.), has taken actions to preserve the identity and diversity of maize landraces and wild relatives. Historically, spatial isolation has been used in seed production to maintain seed purity. Spatial isolation can also be a key component for a strategy to minimize pollen-mediated gene flow in Mexico between transgenic maize and sexually compatible plants of maize conventional hybrids, landraces, and wild relatives. The objective of this research was to generate field maize-to-maize outcrossing data to help guide coexistence discussions in Mexico. In this study, outcrossing rates were determined and modeled from eight locations in six northern states, which represent the most economically important areas for the cultivation of hybrid maize in Mexico. At each site, pollen source plots were planted with a yellow-kernel maize hybrid and surrounded by plots with a white-kernel conventional maize hybrid (pollen recipient) of the same maturity. Outcrossing rates were then quantified by assessing the number of yellow kernels harvested from white-kernel hybrid plots. The highest outcrossing values were observed near the pollen source (12.9% at 1 m distance). The outcrossing levels declined sharply to 4.6, 2.7, 1.4, 1.0, 0.9, 0.5, and 0.5% as the distance from the pollen source increased to 2, 4, 8, 12, 16, 20, and 25 m, respectively. At distances beyond 20 m outcrossing values at all locations were below 1%. These trends are consistent with studies conducted in other world regions. The results suggest that coexistence measures that have been implemented in other geographies, such as spatial isolation, would be successful in Mexico to minimize transgenic maize pollen flow to conventional maize hybrids, landraces and wild relatives.

## Introduction

Cultivation of transgenic crops has been a focus of Mexico’s regulatory framework for agricultural biotechnology since 1996 [1]. In Mexico, there is interest in the agronomic benefits provided by transgenic varieties (*e*.*g*., insect protection, herbicide tolerance) which may result in higher yields and lower cost of production for farmers [2]. However, since Mexico is considered the center of origin and diversity of maize [3], a primary concern of Mexico’s regulatory agencies has been the potential consequences resulting from pollen flow from transgenic maize to native sexually compatible species. Small-scale Mexican farmers typically grow local maize varieties that have been selected for higher yield potential under local biotic and abiotic stresses, appropriate maturity, response to farmers’ management practices, with particular nutrient or culinary properties and storage requirements [4]. However, economic incentive programs and seed exchanges have encouraged small subsistence farmers to replace these varieties that have been bred within their own agroecosystem with improved, introduced cultivars [4–5]. Louette and Smale reported that of the 26 varieties grown by farmers in Cuzalapa, Mexico, only six were local (blanco, negro, amarillo ancho, chianbuiahuitl, tabloncillo and perla). The remaining 20 are classified as introduced (the three major varieties: amarillo, enano and argentino and 17 minor varieties including improved varieties and hybrids) [5]. Most of the introduced varieties were brought in by farmers from surrounding region (less than 100 km away), but some varieties were brought from other world areas (e.g., guino and argentino varieties originated from the USA and Argentina, respectively) [5]. Mexican farmers typically test these new cultivars carrying desirable traits from other regions against their local varieties and adopt those that demonstrate advantage over a number of cropping seasons [5]. To protect genetic resources in Mexico, a special protection regime for maize was described in the 2008 Regulation for the Biosafety Law for Genetically Modified Organisms [6]. The major protection requirements include: 1) establishing isolation zones for areas that are considered centers of origin of maize, and 2) implementing policies for protection, utilization and sustainable use of those species for which Mexico is considered the center of origin and genetic diversity.

In many countries, farmers already have the choice to cultivate non-transgenic and/or approved transgenic varieties and hybrids [7]. Farmers use different production practices to allow crop varieties to effectively coexist in proximity to each other, and each farmer is able to farm according to the economic production standards of their choice (e.g., using or not using transgenic crops), without impeding their neighbor’s ability to make a different choice. From an agricultural perspective, this ‘coexistence’ is the ability to grow crops with different characteristics or intended markets while maintaining intended product integrity and economic value [8]. To enable growers to successfully produce their choice of non-transgenic and transgenic maize, some countries have issued guidance with practical coexistence measures like spatial and/or temporal isolation and possible use of border rows to manage natural cross-pollination rates [8]. For years, similar measures have also ensured maintenance of the appropriate genetic purity standards for seed production and specialty crops [9]. To enable effective, practical and science-based coexistence of diverse maize farming practices in Mexico, there is a need to evaluate the coexistence experience and practical measures successfully utilized elsewhere (*e*.*g*., the USA, Spain). This information can help to confirm that gene flow from transgenic maize can be minimized under Mexican crop-growing conditions [8–10]. Successful coexistence would result in the ability for Mexican growers to chose from diverse farming practices and effectively meet the requirements of consumer and specialty markets, thus ensuring a strong, vibrant, and diverse agricultural economy [11].

Maize varieties/hybrids are characterized by wind-assisted pollination that facilitates outcrossing [12–14]. To minimize undesired outcrossing and to maintain genetic purity, some countries recommend producing transgenic and conventional maize using prescribed isolation distances [15–18]. In addition to distance, other factors that influence outcrossing include: pollen viability and longevity; male fertility or sterility; wind direction and velocity; size, shape, and orientation of fields of pollen source and recipient; flowering synchrony; topography; and vegetation growing between pollen source and pollen receptor fields [19–23]. When coexistence measures are employed together, a combination of isolation distance and border rows can be effective in maintaining the 0.9% threshold established by the European Union [24] and could further reduce the isolation distance to less than 20 m [25–29].

Biosafety guidelines are available that describe the requirements for cultivating transgenic crops in Mexico [6]. Isolation requirements for experimental field trials with transgenic maize have been conservatively set at 250 m, with additional restrictions in regions identified as centers of origin [30]. The objective of this study was to evaluate the outcrossing rates of maize in Mexico compared to those observed in other world regions. This information will be useful to regulators when assessing isolation of conventional maize hybrids or landraces from neighboring fields of transgenic maize. Furthermore, it will provide information useful to assess questions related to the planting of transgenic maize in agricultural regions near centers of origin.

## Materials and Methods

### Site Descriptions

**Fig 1:**
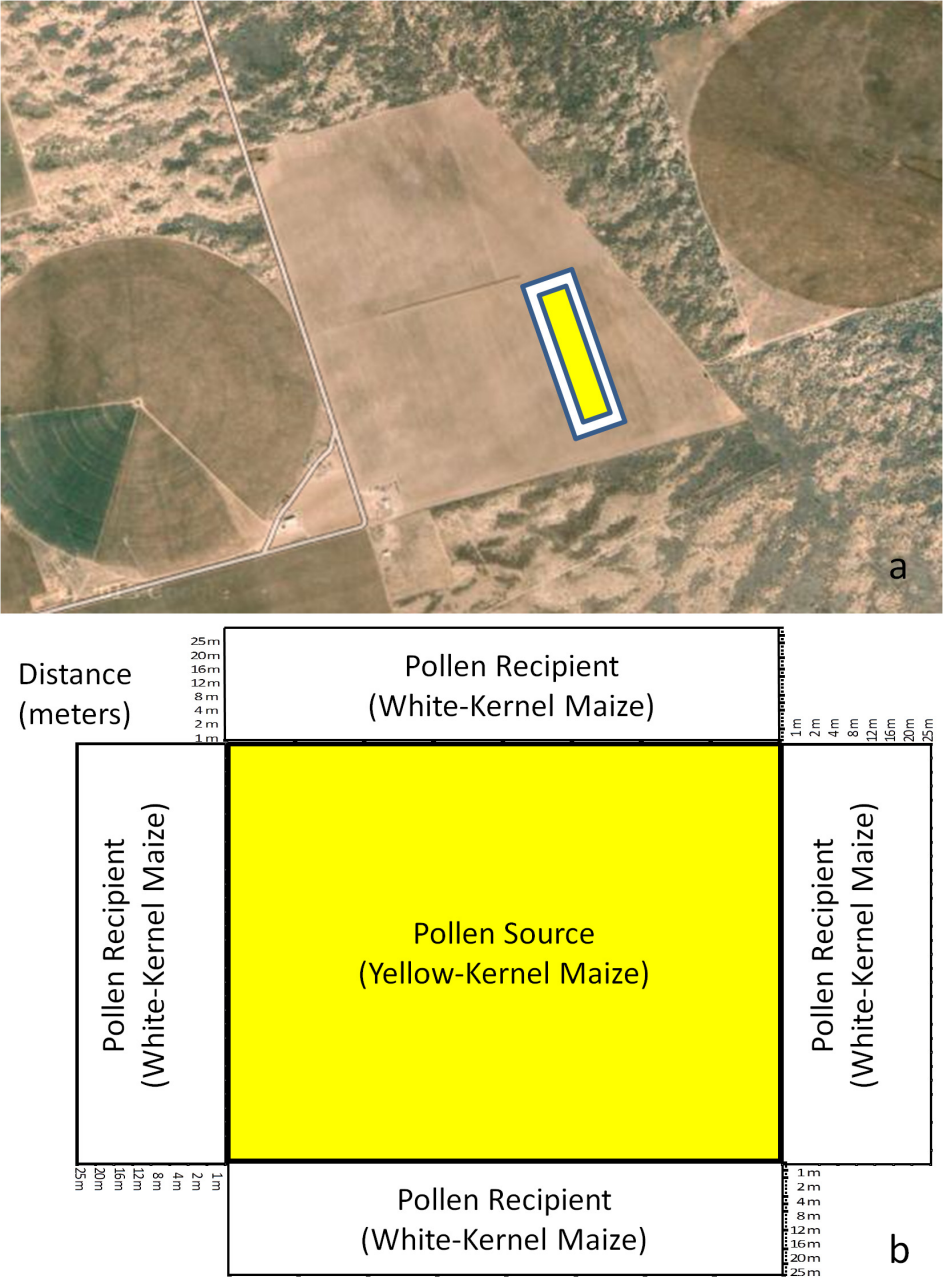
Field layout. (a) An aerial photograph from one of the sites (La Bombas, Chihuahua) and (b) Graphic representation of field layout used across sites to measure outcrossing rates (%) as a function of distance (m).

Field trials were planted at eight locations in northern Mexico. Four sites were planted in 2011 (Mocorito-Pericos, Sinaloa; Ciudad Obregón, Sonora; Las Bombas, Aldama County, Chihuahua; and Francisco I Madero, Coahuila), three sites were planted in 2012 (Ciudad Constitución, Baja California Sur; Valle Hermoso, Tamaulipas; and Culiacancito, Sinaloa), and one site was planted in 2013 (Guasave, Sinaloa) (Table 1). These locations are within the largest hybrid maize growing region in Mexico and are geographically diverse (*e*.*g*., latitudes ranged from 24° to 28°N, longitudes ranged from 97° to 111°W). Trials were conducted in open fields with no sexually compatible crops, fences, or other barriers to alter wind flow within at least 200 m in any direction (Fig 1A). Furthermore, the absence of other maize fields ensured that observed outcrossing was due to pollen flow from the pollen source block. Details regarding the planting dates, pollen source and pollen recipient areas, and geographic coordinates of the eight sites are listed in Table 1.

**Table 1:** Planting dates, pollen source and pollen recipient areas, and geographic coordinates of each study site.

				Coordinates	
City and State	Planting date m/d/y^1^	Pollen source area (ha)	Pollen recipient area (ha)	Latitude (N)	Longitude (W)
Ciudad Constitución, Baja California Sur^2^	03/28/2012	0.5	0.3	25° 0' 36"	111° 39' 48"
Mocorito-Pericos, Sinaloa^2^	03/01/2011	0.2	0.3	24° 59' 51"	107° 40' 23"
Ciudad Obregón, Sonora^2^	03/12/2011	0.4	0.4	27° 23' 2"	110° 2' 41"
Las Bombas, Chihuahua^2^	07/09/2011	0.6	0.3	28° 52' 15"	104° 47' 6"
Francisco I Madero, Coahuila^2^	07/23/2011	0.6	0.4	25° 31' 57''	103° 14' 36''
Valle Hermoso, Tamaulipas^2^	03/18/2012	0.5	0.3	25° 45' 1''	97° 47' 42''
Culiacancito, Sinaloa^3^	01/27/2012	5.0	0.9	24° 49' 13"	107° 32' 39"
Guasave, Sinaloa^3^	01/25/2013	3.9	1.2	25° 40' 43"	108° 35' 14"

^1^ m/d/y = month/day/year.

^2^ Experimental trials.

^3^Pilot trials.

The trials were performed in compliance with the Law on Biosafety for Genetically Modified Organisms and applicable legislation. The considerations for use, mapping and implementation of the field protocols were made with the full consent and assistance of local farmers at each site where the trials were performed. The trials and data collection were conducted by field teams from public academic and research institutions in each region.

### Production Practices

Fields were managed according to the agronomic recommendations of the technical guidelines developed by the National Research Institute for Forestry, Agriculture and Livestock [31]. Agronomic practices typical for each region were applied, including adequate fertilization with phosphorus, nitrogen, and potassium to ensure good quality field trials. Across sites, the average planting population density was 80,000 seeds ha^-1^ with 0.80 m row spacing. All locations were irrigated according to the watering management plan to provide sufficient moisture for optimum crop development.

The specific protection regime for testing transgenic maize in Mexico requires initial evaluation based on small field trials (experimental trials), prior to granting permission for larger, pre-commercial trials (pilot trials). In this study, a total of eight locations planted in northern Mexico were considered (six locations of experimental trials and two locations of pilot trials). Pollen source plots ranged from 0.2 to 0.6 ha for experimental trials and from 3.9 to 5.0 ha for pilot trials (Table 1). Pollen recipient plots were planted as border rows around the pollen source plot (Fig 1A and 1B) and ranged from 0.3 to 1.2 ha depending on the shape and size of the pollen source field (Table 1).

### Pollen Source and Pollen Recipient Materials

Pollen source plots were planted with yellow-kernel transgenic maize corresponding to breeding stacks with the following OECD (Organization for Economic Co-operation and Development) identifiers [32]: MON-89Ø34-3 × MON-88Ø17-3, MON-89Ø34-3 × MON-ØØ6Ø3-6 and/or MON-ØØ6Ø3-6. Maize event MON-89Ø34-3 produces two proteins (Cry1A.105 and Cry2Ab2) that protect against feeding damage caused by lepidopteran insect pests. Cry1A.105 is a modified *Bacillus thuringiensis* (*Bt*) Cry1A protein and Cry2Ab2 is a *Bt* (*B*. *t*. subsp. *kurstaki*) protein. Maize event MON-88Ø17-3 produces a modified *Bt* (*B*. *t*. subsp. *kumamotoensis*) protein, Cry3Bb1, that protects against coleopteran insect pests. In addition, MON-88Ø17-3 also produces the 5-enolpyruvyl-shikimate-3-phosphate synthase protein from *Agrobacterium* sp. strain CP4 (CP4-EPSPS) that confers tolerance to glyphosate. Maize event MON-ØØ6Ø3-6 produces a 5-enolpyruvyl-shikimate-3-phosphate synthase protein from *Agrobacterium* sp. strain CP4 (CP4 EPSPS), which confers tolerance to glyphosate. Pollen recipient plots were planted with commercially-available white-kernel hybrids of the same relative maturity as the pollen source hybrids.

### Measurements

Fields were monitored daily to determine the synchrony of the anthesis and silking periods. The date of anthesis was recorded when 50% of plants had anthers that were shedding pollen. Similarly, the date of silking was recorded when approximately 2–3 cm of silks extended from the tip of the shoot on 50% of plants. Duration of anthesis and silking and the number of number of days that anthesis and silking overlapped was also estimated at each site. During the flowering period, average and maximum daily wind speeds, prevailing wind direction, minimum and maximum daily temperatures, precipitation, as well as relative humidity were collected from the weather station closest to each of the field sites.

At maturity, ears were sampled from the pollen recipient plots in each of the four directions extending from the rectangular pollen source plot (Fig 1B). Measurements were made at the pre-determined distances of 1, 2, 4, 8, 12, 16, 20 and 25 m from the pollen source. These distances were selected based on previous research indicating that the majority of pollen flow occurs in the first few rows adjacent to the source [17]. A total of 30 ears were randomly collected at each of the eight distances and from each of the four different directions around the pollen source field. These ears were marked and bagged individually before counting the number of white and yellow kernels. Empirical outcrossing rates were estimated based on percentage of yellow kernels harvested from the white-kernel hybrid in the pollen recipient plots. Since yellow endosperm is dominant over white, endosperm color was used as visual marker of cross pollination that allowed rapid determination of outcrossing rates.

### Statistical Analysis

A generalized linear mixed model was fit to predict probabilities of outcrossing beyond distances that were observed empirically. The mean outcrossing rates were treated as a continuous response variable confined to the interval (0, 1).

The distribution of such a proportion conditional on a given site was assumed to be *Beta*(*μ*_*ij*_, *φ*), where *μ*_*ij*_ denotes the probability of outcrossing at the *i* ^*th*^ site and *j* ^*th*^ distance. Conceptually, it was presumed that *μ*_*ij*_ varies systematically by distance and is randomly perturbed by variation among sites. Accordingly, to relate the parameter *μ*_*ij*_ to distance, the following linear predictor was defined:

$$\eta_{ij}=\beta_{0}+b_{0i}+\beta_{1}{\text{X}}_{j},$$ (1)

where: β_0_ is the intercept of the regression; *b*_0*i*_ is the random effect of the *i* ^*th*^ *Site* on the intercept of the regression; β_1_ is the slope coefficient; and X_*j*_ is the direct variable reflecting *Distance j*. Note that to improve the fit of the model, the direct variable X_*j*_ was log transformed prior to the analysis. PROC GLIMMIX in SAS Version 9.4 [33] was used to fit the random intercept model (1), employing the logit link function (*i*.*e*., $\eta_{ij}=\text{ln}\;\left(\frac{\mu_{ij}}{1-\mu_{ij}}\right)$).

Maize pollination is wind-assisted [12–14]. Thus, a partial correlation analysis was conducted to quantify the strength of the association between average daily wind speed (km/h) and maximum outcrossing rate (%). Package *ppcor* [34] in R x 64 version 3.0.2 [35] was used for the analysis.

## Results

Pollen source and pollen recipient plants were selected from hybrids of the same relative maturity to ensure synchronous flowering and optimal conditions for outcrossing to occur. At all but one site anthesis and silking of pollen donor and pollen receptor occurred within one day (Table 2). Francisco I Madero, Coahuila was the only site that had three days difference between anthesis and silking, but had nine days of overlap between anthesis of pollen donor and silking of pollen recipient. Across sites, the overlap between anthesis of pollen donor and silking of pollen recipient was on average 9.1 days, which is sufficient to allow for outcrossing.

**Table 2:** Average days to flowering of pollen donor (yellow-kernel maize) and pollen recipient (white-kernel maize) during pollination at each site.

City and State	Pollen donor		Pollen recipient		Anthesis/silking overlap (days)
	Days to anthesis	Duration of anthesis	Days to silking	Duration of silking	
Ciudad Constitución, Baja California Sur	93	12	94	21	11
Mocorito-Pericos, Sinaloa	85	10	84	12	10
Ciudad Obregón, Sonora	80	12	80	7	7
Las Bombas, Chihuahua	49	14	49	7	7
Francisco I Madero, Coahuila	57	12	60	12	9
Valle Hermoso, Tamaulipas	58	11	59	11	10
Culiacancito, Sinaloa	89	12	90	12	11
Guasave, Sinaloa	88	9	89	11	8
**Average**	**74.9**	**11.5**	**75.6**	**11.6**	**9.1**

Both average and maximum wind speed varied widely among the eight locations (Table 3). Average wind speed ranged from 1 km/h at Culiacancito, Sinaloa to 16.5 km/h at Valle Hermoso, Tamaulipas. Maximum wind speed ranged from 4.7 km/h at Ciudad Obregón, Sonora to 23.9 km/h at Ciudad Constitución, Baja California Sur. Direction of prevailing winds during flowering differ depending on location (West for Ciudad Constitution, Baja California Sur; North and West for Mocorito-Pericos and Guasave, Sinaloa; South and West for Culiacancito, Sinaloa; and South and Southeast for Ciudad Obregon, Sonora). No prevailing wind direction was observed for sites Las Bombas, Chihuahua, Francisco I. Madero, Coahuila, and Valle Hermoso, Tamaulipas. There was a wide range of minimum and maximum temperatures observed during flowering across sites (Table 3). Minimum temperatures ranged from 7.7°C at Francisco I Madero, Coahuila to 21.3°C at Valle Hermoso, Tamaulipas. Across sites, maximum temperatures ranged from 26.5°C at Francisco I Madero, Coahuila to 40.1°C at Ciudad Constitucion, Baja California Sur. Precipitation during flowering ranged across sites (Table 3). Minimum or no rain fall (0–1.8 mm) was observed during flowering period for six locations. Two sites, Las Bombas, Chihuahua and Valle Hermoso, Tamaulipas, received a total of 29.3 and 12.4 mm rainfall, respectively (Table 3). Relative humidity ranged across locations with a minimum of 43.8% for Las Bombas, Chihuahua site and a maximum of 77.3% for Valle Hermoso, Tamaulipas site (Table 3).

**Table 3:** Wind speed, prevailing wind direction, average temperatures, precipitation and relative humidity during pollination at each site.

City or Site, State	Wind speed (km/h)		Prevailing wind direction^2^	Average Temperature (°C)		Precipitation (mm)	Relative Humidity (%)
	Average (Range)^1^	Max (Range) ^1^		Min	Max		
Ciudad Constitución, Baja California Sur	10.4 (6.1–17.3)	23.5 (9.7–33.1)	W	14.0	40.1	1.8	63.0
Mocorito-Pericos, Sinaloa	2.7 (1.8–3.9)	19.3 (14.1–27.2)	N, W	15.5	31.1	0.0	61.6
Ciudad Obregón, Sonora	3.2 (2.8–5.3)	4.7 (3.2–7.9)	S, SE	15.6	39.2	0.0	71.0
Las Bombas, Chihuahua	10.9 (8.2–16.6)	21.8 (16.7–29.9)	-^4^	10.4	37.3	29.3	43.8
Francisco I Madero, Coahuila	6.2 (1.8–11.5)	17.6^3^	-	7.7	26.5	0.0	44.7
Valle Hermoso, Tamaulipas	16.5 (12.9–26.3)	23.9 (19.6–36.7)	-	21.3	32.1	12.4	77.3
Culiacancito, Sinaloa	1.0 (0.5–2.0)	14.0 (9.6–20.9)	S, W	15.3	34.7	1.3	61.9
Guasave, Sinaloa	4.9 (3.0–8.5)	23.7 (19.0–30.3)	N, W	12.7	32.7	0.1	62.5

^1^Range of daily wind speed (average and maximum) during flowering.

^2^N, E, W and S = North, East, West and South, respectively.

^3^No data available.

^4^No prevailing winds.

**Fig 2:**
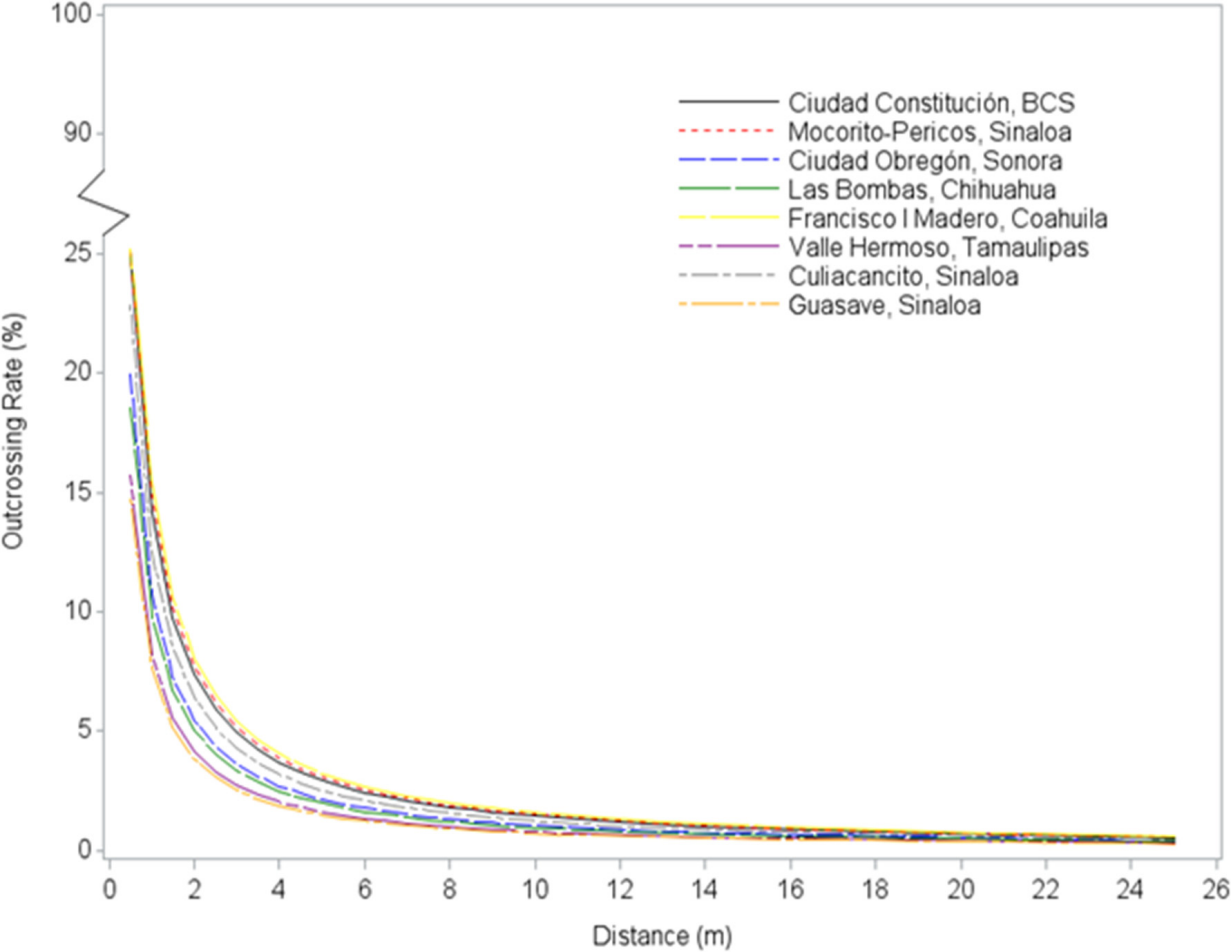
Modeled outcrossing rates (%) per site as a function of distance (m). Note the site-specific equations for predicting outcrossing are shown in Table 5.

Outcrossing rates observed in this study were dependent upon distance of the pollen recipient from the pollen source (Table 4, Fig 2). At all sites, the outcrossing rate was the highest at 1 m from the pollen source (12.9%), and the lowest at 25 m (0.5%). At 1 m, the lowest outcrossing rate (6.4%) was detected at the Guasave, Sinaloa site, and the highest outcrossing rate (21.5%) was observed at the Mocorito-Pericos, Sinaloa site (Table 4). The estimated standard errors provide a measure of how close the sample outcrossing rates are likely to be to the true, underlying means. The small magnitude of these estimates indicates that outcrossing rates were estimated with a high degree of precision.

**Table 4:** Outcrossing rates (%) observed for different distances between pollen source and pollen recipient plots at each site.

City and State	Outcrossing rates at each distance (m)							
	1	2	4	8	12	16	20	25
Ciudad Constitución, Baja California Sur	14.8 (1.73)^1^	6.0 (0.75)	3.5 (0.48)	2.2 (0.37)	1.5 (0.24)	1.1 (0.14)	0.7 (0.12)	0.6 (0.10)
Mocorito-Pericos, Sinaloa	21.5 (2.41)	5.2 (0.71)	2.9 (0.39)	1.5 (0.22)	1.1 (0.14)	0.6 (0.08)	0.5 (0.08)	0.4 (0.07)
Ciudad Obregón, Sonora	10.3 (1.45)	4.6 (0.59)	2.5 (0.28)	1.4 (0.14)	0.9 (0.11)	0.7 (0.12)	0.7 (0.09)	0.7 (0.12)
Las Bombas, Chihuahua	9.5 (1.64)	4.0 (0.84)	1.3 (0.41)	0.9 (0.16)	0.7 (0.18)	0.8 (0.22)	0.5 (0.14)	0.3 (0.07)
Francisco I Madero, Coahuila	19.5 (1.99)	6.1 (0.67)	3.7 (0.43)	1.6 (0.22)	1.2 (0.17)	0.7 (0.10)	0.6 (0.12)	0.7 (0.15)
Valle Hermoso, Tamaulipas	6.5 (0.79)	3.1 (0.33)	2.8 (0.35)	1.5 (0.14)	0.8 (0.12)	0.7 (0.11)	0.4 (0.08)	0.6 (0.07)
Culiacancito, Sinaloa	14.6 (1.43)	5.3 (0.45)	2.8 (0.30)	1.2 (0.13)	0.8 (0.10)	0.7 (0.08)	0.5 (0.06)	0.5 (0.06)
Guasave, Sinaloa	6.4 (0.60)	2.7 (0.37)	2.0 (0.24)	1.1 (0.14)	0.9 (0.16)	0.7 (0.12)	0.5 (0.07)	0.4 (0.10)
**Average Outcrossing (%)**	**12.9 (0.59)**	**4.6 (0.22)**	**2.7 (0.13)**	**1.4 (0.07)**	**1.0 (0.06)**	**0.8 (0.05)**	**0.5 (0.03)**	**0.5 (0.03)**
**Total number of ears** ^**2**^	**945**	**948**	**942**	**949**	**950**	**950**	**1070**	**950**

^1^Mean and Standard Errors.

^2^Total number of ears sampled across all sites at each distance.

**Fig 3:**
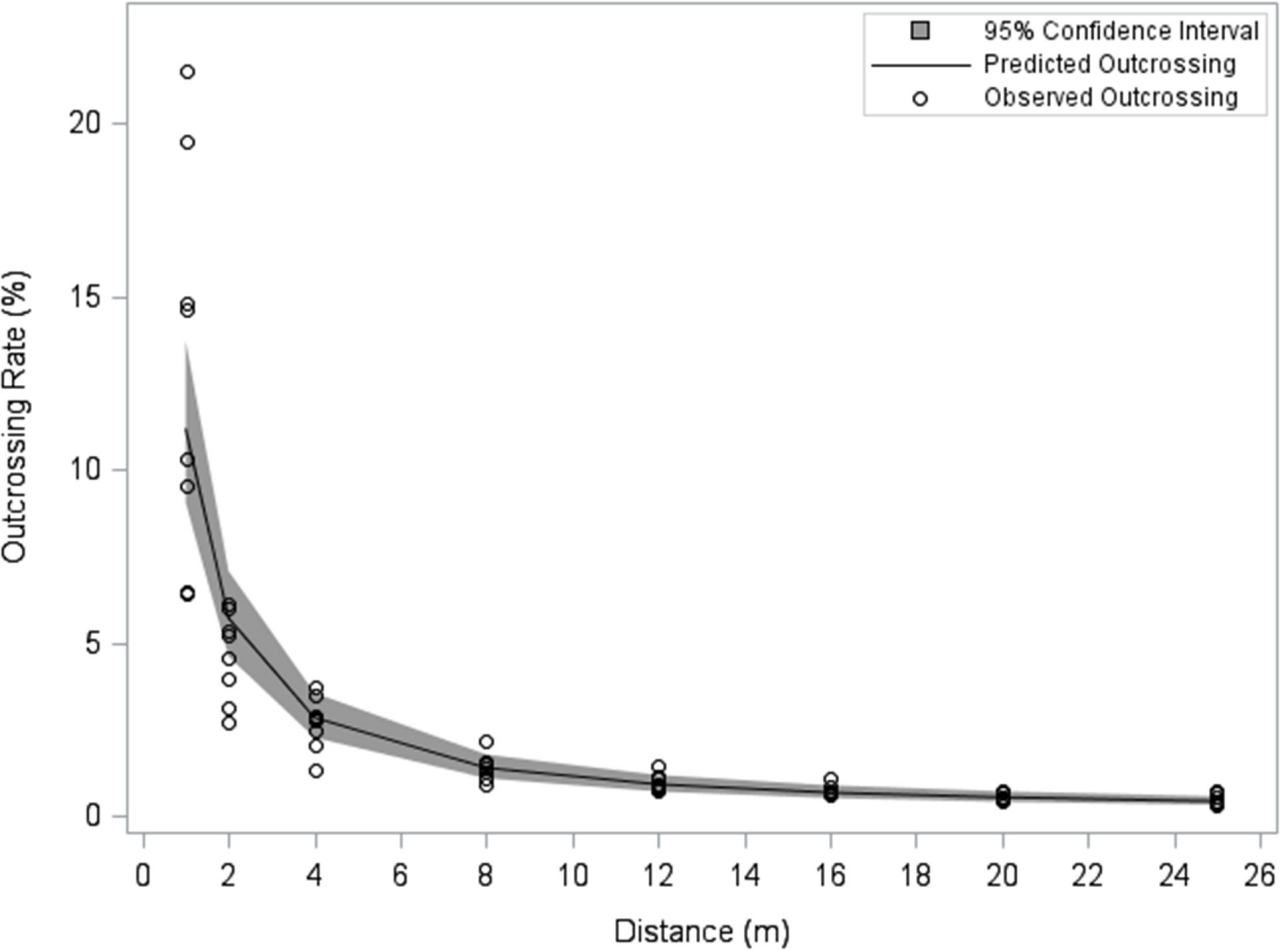
Observed and predicted outcrossing rates (%) across sites as a function of distance (m). Observed values (circles) and predicted values (line) based on model (1) with 95% confidence interval (shaded region).

**Fig 4:**
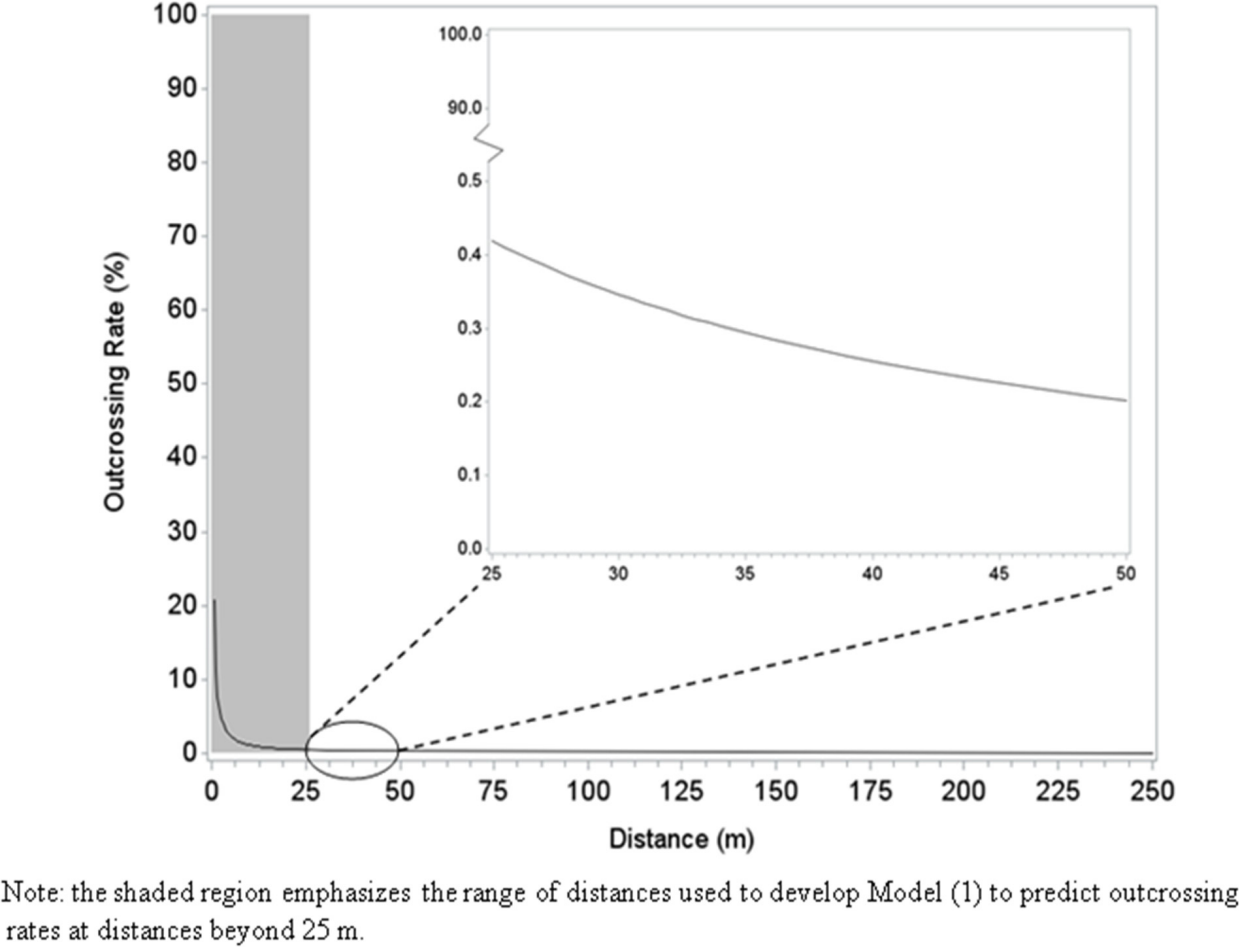
Predicted outcrossing rates (%) for distances beyond 25 m.

At the maximum measured distance (25 m), the outcrossing rate was the lowest (0.3%) at the Las Bombas, Chihuahua site and the highest (0.7%) at the Ciudad Obregón, Sonora and the Francisco I Madero, Coahuila sites. Regardless of numerical differences observed across sites, the decline in outcrossing with increased distance was comparable and qualitatively consistent across locations (Fig 2). There was little among-site variation beyond 12 meters, where 28 of 32 observed outcrossing values were less than 1%. In this study, all outcrossing values were less than 1% at 20 and 25 m from the pollen source. The large majority of pollen deposition, and the greatest variability among sites, occurred within the first two meters from the pollen source. The predicted values from the model (1) reflected well the observed outcrossing values (Fig 3). The estimated regression equations (Table 5) can be used to predict across site averages, as well as site-specific outcrossing rates at particular distances. These results indicated that the average outcrossing rate is expected to be less than 0.2% beyond 50 meters (Fig 4).

**Table 5:** Site-specific equations for predicting outcrossing rates based on model (1).

City and State	Model Equation
Ciudad Constitución, Baja California Sur	Outcrossing Rate = $\frac{e^{-\left(1.80+1.06*\text{ln}\left(Distance\right)\right)}}{1+e^{-\left(1.80+1.06*\text{ln}\left(Distance\right)\right)}}$
Mocorito-Pericos, Sinaloa	Outcrossing Rate = $\frac{e^{-\left(1.75+1.06*\text{ln}\left(Distance\right)\right)}}{1+e^{-\left(1.75+1.06*\text{ln}\left(Distance\right)\right)}}$
Ciudad Obregón, Sonora	Outcrossing Rate = $\frac{e^{-\left(2.13+1.06*\text{ln}\left(Distance\right)\right)}}{1+e^{-\left(2.13+1.06*\text{ln}\left(Distance\right)\right)}}$
Las Bombas, Chihuahua	Outcrossing Rate = $\frac{e^{-\left(2.31+1.06*\text{ln}\left(Distance\right)\right)}}{1+e^{-\left(2.31+1.06*\text{ln}\left(Distance\right)\right)}}$
Francisco I Madero, Coahuila	Outcrossing Rate = $\frac{e^{-\left(1.70+1.06*\text{ln}\left(Distance\right)\right)}}{1+e^{-\left(1.70+1.06*\text{ln}\left(Distance\right)\right)}}$
Valle Hermoso, Tamaulipas	Outcrossing Rate = $\frac{e^{-\left(2.41+1.06*\text{ln}\left(Distance\right)\right)}}{1+e^{-\left(2.41+1.06*\text{ln}\left(Distance\right)\right)}}$
Culiacancito, Sinaloa	Outcrossing Rate = $\frac{e^{-\left(1.95+1.06*\text{ln}\left(Distance\right)\right)}}{1+e^{-\left(1.95+1.06*\text{ln}\left(Distance\right)\right)}}$
Guasave, Sinaloa	Outcrossing Rate = $\frac{e^{-\left(2.49+1.06*\text{ln}\left(Distance\right)\right)}}{1+e^{-\left(2.49+1.06*\text{ln}\left(Distance\right)\right)}}$
**Across Sites**	**Outcrossing Rate =** $\frac{e^{-\left(2.07+1.06*\ln\left(Distance\right)\right)}}{1+e^{-\left(2.07+1.06*\ln\left(Distance\right)\right)}}$

A particular transgene present in a maize hybrid is typically introduced via only one of the inbred parents, resulting in hemizygous hybrids and only 50% of the hybrid’s pollen carrying the transgene [16]. If this transgene hemizygosity assumption is applied to the outcrossing data presented here, all estimates will be lower for gene flow associated with transgenic maize hybrids. Specifically, for a single transgene the outcrossing rate will be reduced by 50%; beyond 25 m estimated to be less than 0.2% and beyond 50 m estimated to be less than 0.1%. However, if two or more unlinked transgenes are considered, then the transgenic gene flow values as predicted by the model beyond 25 m would be between 0.4 and 0.2%; and beyond 50 m would be between 0.2 and 0.1%.

Considering that maize is a wind-pollinated species [12–14], the association between outcrossing rate and wind speed was assessed. A partial correlation analysis was conducted to quantify the strength of the association between average daily wind speed (km/h) and maximum outcrossing rate (%), while controlling for distance. The estimated correlation coefficient was 0.01 (p = 0.93, n = 64) indicating lack of association between outcrossing rate and wind speed in this study.

## Discussion

Mexico is considered the center of origin of maize; as such, coexistence measures must be evaluated for diverse agronomic production systems using modern transgenic and non-transgenic maize hybrids, as well as the preservation of traditional landraces. Farming systems based on cultivating transgenic and non-transgenic hybrids already effectively coexist in other geographies [25–27]. Science-based information on practical coexistence measures can inform policies designed to ensure that Mexican maize growers have equal access to the modern or traditional farming practices of their choice. Such policies can also ensure meeting standards for production and integrity of regional landraces.

Coexistence measures, such as borders rows or physical distance from the pollen source, can be used to minimize outcrossing; a) from transgenic maize to conventional maize and landraces, and b) from conventional hybrids to landraces. Coexistence-enabling measures for maize generally rely on science-based recommendations for either temporal or spatial isolation between pollen donors and pollen recipients to minimize the potential for outcrossing [36]. In this study, data was generated to contribute to a better understanding of spatial isolation. Acceptable flowering synchrony was achieved, thus eliminating conditions of temporal isolation (*i*.*e*. offset anthesis and silking timing between pollen donor and recipient, respectively) [37–39]. The intentional flowering synchrony in this study, therefore, has resulted in highly conservative outcrossing rates and an overestimation of what would be experienced by most farmers.

Results from this study indicate that outcrossing rates are dependent upon distance from the pollen source. Similar observations have been reported for numerous trials conducted in different world regions [17], [21–23], [25–26], [38], [40–45]. For comparison and simplicity, the reported outcrossing data from each study were averaged over the following distance ranges (1–5, 5–10, 10–25, 25–50, 50–100 m and over 100 m) from the pollen source (Table 6).

**Table 6:** Outcrossing rates (%) reported in different world regions and compared to observed or predicted outcrossing rates in this study.

Country	Distances^1^					
	1–5 m	5–10 m	10–25 m	25–50 m	50–100 m	>100 m
US[21–22], [40], [44–45],	6.5–30.1	1.5	0.8–2.4	0.4–0.6	0.2	0.0
Mexico[23], [38]	17.0	3.7	0.9	0.2	-	0.0
Canada[17]	14.4	2.0	0.6	0.2	-	-
Spain[26–27]	6.9–7.0	2.4–5.2	1.7–1.9	0.5	0.1–0.4	0.0
UK[41]	20.5	5.6	0.8	0.3	-	-
Switzerland[42]	-	-	-	-	0.0	0.0
China[43]	14.5	9.3	1.7	0.6	0.2	-
**Overall range across world regions**	**6.5–30.1**	**1.5–9.3**	**0.6–2.4**	**0.2–0.6**	**0.0–0.4**	**0.0**
**Average values from this study** ^**2**^	**6.7**	**1.4**	**0.7**	**0.5**	**0.1–0.3**	**<0.1**

^1^Excluding the maximum distance for each range (*e*.*g*., 5 m distance is included in 5–10 m range, but excluded from 1–5 m range).

^2^Average observed values (for 1–5 m, 5–10 m, 10–25 m, and 25–50 m distances) and estimated values based on predicted model (for 50–100 m and >100 m distances).

Note: Outcrossing values reported by Messeguer *et al*. were averaged over fields and distances within the range.

Also, reported values were doubled to adjust for hemizygosity of tested material [26]. Outcrossing values reported by Bateman were averaged over fields and distances within the range [41].

The data presented in Table 6 across geographies illustrates that a) most outcrossing was observed within the first 5 m from the pollen source, b) outcrossing declined sharply with distance, and c) outcrossing dropped below 1% after 25 m. The results described in Table 6 are similar to the results observed in the present study in which a) most outcrossing was observed near the pollen source (6.7% at 1–5 m), b) outcrossing declined sharply to 1.4% at 5–10 m, and c) the outcrossing rates were below 1% after 20 m (Table 4). Furthermore, the majority of outcrossing variation observed in the present study was for distances closest to the pollen source (Table 4), which was also true for data summarized across world regions (Table 6). Very little variation across sites and regions was observed at distances greater than 20 m from the potential pollen donor.

In the present study, the lack of correlation (0.01) between wind speeds and percent outcrossing across sites indicate that wind speed may not contribute significantly to outcrossing rates. There was a large variation in both the average and the maximum daily wind speeds (1.0–17.5 km/h and 4.7–26.3 km/h, respectively) across sites (Table 3); the sites with the highest wind speeds, however, were not associated with the most outcrossing. Ma *et al*. also observed lack of positive association between the wind speed and outcrossing rate [17]. In their study, fields with average wind speed of 1–5 km/h showed 18.2% outcrossing rate, whereas those with average wind speed of 5–12 km/h had 13.3% outcrossing rate in the rows adjacent to pollen source. Likewise, Weber *et al*. noted that the influence of wind can vary between sites and years, so that a reliable prediction is not possible [28]. Thus, wind speed and direction cannot be reliably incorporated in strategies to avoid cross-pollination [28].

The outcrossing values estimated in the present study (Fig 4) are in general agreement with empirical work conducted by others. Luna *et al*. studied gene flow in Mexico and found only one outcrossed kernel at each distances of 100, 150, and 200 m from the pollen source, and no outcrossing at greater distances (300 and 400 m) [23]. In other study published by Cervantes in Mexico, no outcrossing beyond 32 m from the pollen source was detected [38]. Raynor *et al*. conducted a field study in the USA and estimated that less than 1% of pollen grains would travel beyond 60 m [37], which is not unexpected considering that maize pollen is the largest and heaviest among the *Poaceae* wind-pollinated species [46–47], with pollen grain sizes ranging from 103 and 105 μm in diameter [13] and settling velocity of 0.2 to 0.3 m/sec [20].

The consequences of gene movement in maize have been well understood and effectively managed for decades. A well recognized example in Mexico is the cultivation of conventional maize hybrids and improved open pollinated varieties in close proximity to landraces of maize. Conventional maize hybrids have coexisted with landraces in traditional agricultural systems with no dramatic displacement of landraces [48]. Since the outcrossing potential for transgenic and conventional maize hybrids is no different, the effect of transgenic maize presence on landrace diversity should not differ from that observed with the inclusion of elite maize hybrids into traditional systems [48–49].

Effective management of outcrossing in maize is shown via the field standards used for production of maize pre-foundation seed, foundation seed, and hybrid seed [36]. In each case, spatial isolation is recognized as an effective measure to maintain certain purity levels and is sometimes modified by the use of natural barriers, differential maturity dates, or male sterile parents. Based on ouctrossing values in this study and outcrossing rate values across geographies presented in Table 6, it is concluded that 20 m isolation distance is sufficient to have outcrossing levels under 1%. If less than 0.1% of outcrossing is required, distances beyond 100 m are recommended (Table 6).

The data presented here demonstrate that spatial isolation is an effective method for reducing outcrossing rates from transgenic maize to conventional maize or landraces in Mexico. Since landraces are not typically commercially grown in northern Mexico, the probability of outcrossing with transgenic maize is minimal. Spatial isolation in conjunction with other methods (*e*.*g*., temporal isolation) will significantly mitigate concerns derived from the planting of transgenic maize in the main hybrid maize production regions in northern Mexico.
